# RETRACTION: “Effects of Crude Extracts from Medicinal Herbs *Rhazya stricta* and *Zingiber officinale* on Growth and Proliferation of Human Brain Cancer Cell Line In Vitro”

**DOI:** 10.1155/bmri/9807398

**Published:** 2026-04-06

**Authors:** BioMed Research International

RETRACTION: A. I. Elkady, R. A. El Hamid Hussain, and O. A. Abu‐Zinadah, “Effects of Crude Extracts from Medicinal Herbs *Rhazya stricta* and *Zingiber officinale* on Growth and Proliferation of Human Brain Cancer Cell Line In Vitro,” *BioMed Research International,* vol. 2014 (2014). https://doi.org/10.1155/2014/260210.

The above article, published online on 22 July 2014 in Wiley Online Library (wileyonlinelibrary.com), has been retracted by agreement between the journal’s Section Editor, Jinsong Ren; and John Wiley & Sons Ltd. The retraction has been agreed following an investigation of the concerns raised by *Mycosphaerella arachidis* and *Orthodicranum flagellare* on PubPeer [1], which identified several concerns related to the figures in the paper. More specifically, multiple panels in Figure 2b share similar features, despite belonging to different treatment groups. As a result of the investigation, the data and conclusions of this article are considered unreliable, therefore the article must be retracted.

The authors have been informed of this decision but did not provide a response.
